# DNA barcoding of *Oryza*: conventional, specific, and super barcodes

**DOI:** 10.1007/s11103-020-01054-3

**Published:** 2020-09-03

**Authors:** Wen Zhang, Yuzhe Sun, Jia Liu, Chao Xu, Xinhui Zou, Xun Chen, Yanlei Liu, Ping Wu, Xueying Yang, Shiliang Zhou

**Affiliations:** 1 State Key Laboratory of Systematic & Evolutionary Botany, Institute of Botany, Chinese Academy of Sciences, Beijing, 100093 China; 2 College of Life Sciences, University of Chinese Academy of Sciences, Beijing, 100049 China; 3 College of Landscape Architecture, Northeast Forestry University, Haerbin, 150040 China; 4 College of Life Science, Sichuan Agricultural University, Yaan, 625014 Sichuan China; 5 Key Laboratory of Forensic Genetics, Institute of Forensic Science, Ministry of Public Security, China, Beijing, 100038 China

**Keywords:** Chloroplast genome, DNA barcode, *Oryza*, Phylogenomics, Seed identification

## Abstract

**Key message:**

We applied the phylogenomics to clarify the concept of rice species, aid in the identification and use of rice germplasms, and support rice biodiversity.

**Abstract:**

Rice (genus *Oryza*) is one of the most important crops in the world, supporting half of the world’s population. Breeding of high-yielding and quality cultivars relies on genetic resources from both cultivated and wild species, which are collected and maintained in seed banks. Unfortunately, numerous seeds are mislabeled due to taxonomic issues or misidentifications. Here, we applied the phylogenomics of 58 complete chloroplast genomes and two hypervariable nuclear genes to determine species identity in rice seeds. Twenty-one *Oryza* species were identified. Conspecific relationships were determined between *O. glaberrima* and *O. barthii*, *O. glumipatula* and *O. longistaminata*, *O. grandiglumis* and *O. alta*, *O. meyeriana* and *O. granulata*, *O. minuta* and *O. malampuzhaensis*, *O. nivara* and *O. sativa* subsp. *indica*, and *O. sativa* subsp. *japonica* and *O. rufipogon.* **D** and **L** genome types were not found and the **H** genome type was extinct. Importantly, we evaluated the performance of four conventional plant DNA barcodes (*matK*, *rbcL*, *psbA-trnH*, and ITS), six rice-specific chloroplast DNA barcodes (*psaJ*-*rpl33*, *trnC-rpoB*, *rps16*-*trnQ*, *rpl22-rps19*, *trnK*-*matK*, and *ndhC-trnV*), two rice-specific nuclear DNA barcodes (NP78 and R22), and a chloroplast genome super DNA barcode. The latter was the most reliable marker. The six rice-specific chloroplast barcodes revealed that 17% of the 53 seed accessions from rice seed banks or field collections were mislabeled. These results are expected to clarify the concept of rice species, aid in the identification and use of rice germplasms, and support rice biodiversity.

**Electronic supplementary material:**

The online version of this article (10.1007/s11103-020-01054-3) contains supplementary material, which is available to authorized users.

## Introduction

The last 50 years witnessed an explosion in the human population, which has been supported by a three-fold global expansion in crop production (Tayyib 2013). Rice, maize, and wheat, together with some other staple crops, have been key for this expansion. The rapid increase in crop production has been achieved largely through higher yields per unit and crop intensification. Creation of higher-yielding crop varieties requires specific genes from the gene pool of the crop species and/or its close relatives, such as the semidwarfing gene in rice (*sd-1*) and *Rht1* and *Rht2* in wheat (Gale and Marshall 1973; Jennings 1964). Genetic resources are fundamental for cultivar improvement; however, most crops have suffered a loss of genetic diversity following prolonged domestication. For example, bread wheat, which originated some 8000 years ago in the Fertile Crescent, has undergone several rounds of genetic erosion (Jia et al. 2013). Genetic resources of crops and their close relatives were initially conserved ex situ in seed banks worldwide and later in situ in their homelands or nearby areas. With intense reclamation of arable land, more and more wild forms of crops and their close relatives have been lost, increasing our reliance on germplasms housed in seed banks. However, seeds in seed banks may be mislabeled due to (1) incorrect species taxonomy, (2) lack of diagnostic morphological parameters, and (3) contamination with old material. Therefore, authentication of specimens is crucial to avoid compromising research and crop production. Given that it is not easy to identify seeds based solely on morphology, DNA barcoding has come to offer a promising solution for discriminating between very similar materials.

First proposed in 2003 (Hebert et al. 2003), DNA barcoding has become a reliable technology to rapidly identify species based on short DNA fragments. In 2009, the two-locus combination of *matK* + *rbcL* was recommended as a core barcode for the identification of land plants (Hollingsworth et al. 2009). Following their first mention in 2005 (Kress et al. 2005), internal transcribed spacer of ribosomal DNA (ITS)/ITS2 and *psbA-trnH* were proposed as new barcodes for land plants (Chen et al. 2010; Li et al. 2011; Yan et al. 2015). A region of *ycf1* was also proposed as a barcoding target owing to its high resolution (Dong et al. 2015). Due to unsatisfactory resolution of a single marker in discriminating between species, various combination schemes were assessed (Hollingsworth et al. 2009). Nowadays, the technique is successfully used to discover cryptic species (Huemer et al. 2014; Kress et al. 2009), detect illegally traded, invasive or endangered species (Lahaye et al. 2008), assess biodiversity (Sonstebo et al. 2010), and identify medicinal plants in mixtures (Howard et al. 2012). Despite these and other advancements, conventional DNA barcodes do not work in the case of extremely closely related species or only slightly diverged “species” from a recent radiation event (Hollingsworth et al. 2011). To address such instances, a DNA super barcode was proposed (Li et al. 2015). A DNA super barcode includes a complete genome or parts of a genome containing enough information to discriminate between the species of interest. The entire chloroplast or mitochondrial genomes, combinations of many genes (or regions in a genome), and assemblies of single nucleotide polymorphisms constitute examples of DNA super barcodes. With the advent of super barcodes, seeds of closely related species in seed banks can be finally assigned to the correct species or even individual haplotypes. Rice seeds require super barcodes, such as the entire chloroplast genome, to distinguish between **A** and **C** haploid genome types, which are so closely related that they cannot be resolved using common chloroplast gene fragments.

Rice belongs to the genus *Oryza* in the family Poaceae. The genus consists of about 26 species distributed across tropical and subtropical areas (Vaughan 1989) (Table S1). However, disputes remain regarding the relationship between *O. granulata* and *O. meyeriana*, and between *O. schweinfurthiana* and *O. punctata*. *Oryza* has a very short evolutionary history. It diverged from *Leersia* some 14 million years ago (Guo and Ge 2005) and includes eight known haploid genome types (**A**, **B**, **C**, **E**, **F**, **G**, **J**, **K**, and **L**) and two unknown genome types (**D** and **H**) (Aggarwal et al. 1999). The genus has been subjected to several taxonomic revisions but some issues persist (Liu et al. 2016; Lu et al. 2001; Rougerie et al. 2014; Vaughan 1989). For example, the two subspecies of the Asian rice (*O. sativa*), subsp. *indica* and subsp. *japonica*, are taxonomically incorrect according to International Code of Nomenclature for algae, fungi, and plants (https://www.iapt-taxon.org/nomen/main.php). Akin to African rice (*O. glaberrima*), its accessions are intermingled genetically with those of its wild progenitor (*O. barthii*, Choi et al. 2019; Li et al. 2011).

Cultivated rice is one of the most important cereal crops worldwide and it feeds more than half of the world’s population (Khush 2005). Its wild progenitors or relatives represent precious genetic resources for rice breeding and genetic improvement (Vaughan et al. 2003; Wing et al. 2005) Established genomic tools for the molecular and genetic study of *O. sativa* (Kim et al. 2008; Tang et al. 2010) can facilitate the correct characterization of seeds and the use of genetic resources housed in seed banks. Here, we demonstrate the effectiveness of a rice chloroplast genome super barcode for identifying rice seeds from seed banks. By employing some nuclear DNA barcodes, we also address possible faults of using the rice chloroplast genome super barcode.

## Materials and methods

### Seed acquisition

Fifty-three seed accessions, including two accessions of *Leersia*, were acquired from seed banks or collected from the field (Table 1). They proceeded mostly (41 accessions) from the International Rice Research Institute in the Philippines. Six accessions could not be traced to a particular source and three accessions were collected during our field expedition. Voucher specimens of these samples were deposited in the herbarium of the Institute of Botany, Chinese Academy of Sciences. Based on their names or field identification, the rice samples belonged to 25 species.

**Table 1:** Plant materials of *Oryza* sampled in this study with *Leersia* species as outgroups

	Original name	Source	Accession number	Voucher
1	*Leersia perrieri*	Madagascar	IRGC105164	BOP022686
2	*Leersia tisserantti*	Guinea	IRGC101384	BOP022687
3	*Oryza alta*	Suriname	IRGC100967	BOP022645
4	*Oryza alta*	Guyana	IRGC105143	BOP022646
5	*Oryza australiensis*	Australia	IRGC101410	BOP022647
6	*Oryza australiensis*	Australia	IRGC103303	BOP022648
7	*Oryza australiensis*	Australia	IRGC105277	BOP022649
8	*Oryza barthii*	Mali (Sudan)	IRGC100933	BOP022650
9	*Oryza barthii*	Guinea	IRGC106194	BOP022651
10	*Oryza barthii*	Sierra Leone	IRGC106234	BOP022652
11	*Oryza brachyantha*	Sierra Leone	IRGC105151	BOP022653
12	*Oryza brachyantha*		99-8813	BOP022654
13	*Oryza eichingeri*	Sri Lanka	IRGC81804	BOP022655
14	*Oryza eichingeri*	Uganda	IRGC105159	BOP022656
15	*Oryza eichingeri*			BOP204879
16	*Oryza glumipatula*	Venezuela	IRGC103812	BOP022657
17	*Oryza grandiglumis*	Brazil	IRGC105669	BOP022694
18	*Oryza grandiglumis*	Brazil	IRGC101405	BOP022695
19	*Oryza granulata*	Sri Lanka	IRGC100880	BOP022659
20	*Oryza granulata*	Vietnam	IRGC106469	BOP022660
21	*Oryza granulata*		M9-32	BOP022661
22	*Oryza latifolia*	Costa Rica	IRGC100167	BOP022662
23	*Oryza latifolia*		99-9038	BOP022663
24	*Oryza latifolia*			BOP204878
25	*Oryza longiglumis*	Indonesia	IRGC105146	BOP022664
26	*Oryza longiglumis*	Indonesia	IRGC105148	BOP022665
27	*Oryza longiglumis*	Papua New Guinea	IRGC106525	BOP022666
28	*Oryza malampuzhaensis*			BOP204667
29	*Oryza meridionalis*	Australia	IRGC105281	BOP022667
30	*Oryza meridionalis*	Australia	IRGC105289	BOP022668
31	*Oryza meyeriana*			BOP204877
32	*Oryza minuta*	Philippines	IRGC105126	BOP022669
33	*Oryza minuta*		p90-12	BOP022670
34	*Oryza neocalidonia*	New Caledonia	IRGC89143	BOP022671
35	*Oryza nivara*	Nepal	Ge-NEP0201	BOP022698
36	*Oryza nivara*	Laos	Ge-VN0102	BOP022699
37	*Oryza officinalis*	Bangladesh	IRGC102460	BOP022672
38	*Oryza officinalis*	India	IRGC104708	BOP022673
39	*Oryza officinalis*	Philippines	IRGC105085	BOP022674
40	*Oryza officinalis*	Philippines	IRGC80773	BOP022700
41	*Oryza punctata*	Chad	IRGC105607	BOP022675
42	*Oryza punctata*	Cameroon	IRGC105984	BOP022676
43	*Oryza punctata*	India	IRGC100125	BOP022677
44	*Oryza punctata*	Nigeria	IRGC104059	BOP022678
45	*Oryza punctata*	Zaire	IRGC105137	BOP022679
46	*Oryza rhizomatis*			BOP204880
47	*Oryza ridleyi*	Malaysia	IRGC100877	BOP022680
48	*Oryza ridleyi*	Thailand	Ge-09101	BOP022681
49	*Oryza rufipogon*	Cambodia	IRGC105738	BOP022682
50	*Oryza rufipogon*	Laos	Ge-VN0219	BOP022696
51	*Oryza rufipogon*			BOP022697
52	*Oryza schlechteri*	Papua New Guinea	IRGC82047	BOP022683
53	*Porteresia coarctata*	Bangladesh	IRGC104502	BOP022690

### DNA extraction and chloroplast genome determination

Seedlings were raised from seeds in a greenhouse, harvested, and quickly dried in a convection oven at 65 °C to denature DNAase. Total genomic DNA (~ 30 mg) was extracted from dry leaves using the mCTAB method (Li et al. 2013). A library was constructed and sequenced for each sample at Beijing Novogene Bioinformatics Technology Co., Ltd, Beijing, using an Illumina HiSeq X Ten platform. Chloroplast genome reads were sorted out and the genomes were assembled de novo using SPAdes 3.9 (Bankevich et al. 2012). The generated contigs were mapped to the closest references by blastn 2.8.10 (Altschul et al. 1990), assembled with Sequencher 5.4 (Corperation)and gaps were filled by Sanger sequencing using primers reported by Dong et al. (2013).

### Rice-specific DNA barcode design

Nucleotide diversity across all chloroplast genomes from all *Oryza* species was quantified using DnaSP (Librado and Rozas 2009). The most hypervariable regions were selected as rice-specific barcodes. Primers were designed to amplify and sequence these regions.

To determine the origins of polyploid species, two highly variable and single-copy nuclear genes were selected from 142 candidate genes (Zou et al. 2008). Fragments were amplified using specific primers. The fragments of the same sample were mixed with the chloroplast fragments and sequenced together on an Illumina HiSeq X Ten platform. Reads were extracted using known references and assembled with Sequencher 5.4.

### PCR amplification and sequencing of rice-specific DNA barcodes

The PCR reaction mixture contained 1 × Taq buffer with Mg^2+^, 0.1 mM dNTPs, and 20 ng DNA. The PCR program included 40 cycles at 94 °C for 30 s, 55 °C for 30 s, and 72 °C for 2 min. PCR products were cleaned using PEG8000 and sequenced in both directions on an ABI 3730xl DNA Analyzer (Applied Biosystems). The sequences were assembled using Sequencher 5.4 and edited if necessary to correct some nucleotide calling mistakes.

### Dataset preparation

The newly determined chloroplast genomes (Table S2) were combined with 37 chloroplast genomes (together with chloroplast fragments of three species) downloaded from GenBank (Table S3), aligned using mafft-win (Katoh and Standley 2013), and adjusted manually using Se–Al. Species delimitation, resolution comparison, and seed identification were performed with corresponding datasets using phylogenetic methods.

Dataset 1 contained 58 chloroplast genomes, representing all rice species (1–3 per species), together with three *Leersia* species as outgroups. Maximum parsimony analyses were carried out to identify and exclude mislabeled genomes (wrong systematic positions) or genomes of relatively low quality (longer branch lengths). This dataset was used to delimit the circumscription of species together with dataset 6 and a super barcode of *Oryza*.

Dataset 2 (*matK*), dataset 3 (*rbcL*), dataset 4 (*psbA-trnH*), and dataset 5 (ITS) represented conventional DNA barcodes. The *psbA-trnH* sequence is interrupted by *rps19* in Poaceae. Dataset 6 represented the concatenation of two single-copy nuclear genes (N78 and R22) selected from 142 genes (Zou et al. 2008). The datasets were analyzed using phylogenetic methods to test the resolution of these candidate DNA barcodes. Dataset 7 was formed by the concatenation of six rice-specific chloroplast DNA barcodes identified in this study. This dataset was analyzed using phylogenetic methods for reliable species identification of rice seeds.

### Phylogenetic analyses

#### Maximum parsimony

Maximum parsimony analysis was executed using PAUP version 4.0a150 (Swofford 2003). The tree search used a heuristic strategy with random stepwise addition of 100 replicates, tree bisection and reconnection branch swapping, and saving multiple trees with no more than two tree scores ≥ 5 from each replicate. Branch support for the maximum parsimony trees was assessed with 1000 bootstrap replicates. The trees were rooted using *Leersia* species as outgroups.

#### Maximum likelihood

Maximum likelihood analyses were performed using RAxML (Stamatakis 2014) with the GTR + I + G model. Branch support for the ML trees was assessed with 1000 bootstrap replicates. The trees were rooted using *Leersia* species as outgroups.

#### Bayesian inference

The best-fit substitution models were GTR + I + G and Blosum + I + G selected by running ModelFinder (Kalyaanamoorthy et al. 2017) for dataset 1 and dataset 6. Bayesian inference was assessed with MrBayes 3.2 (Fredrik et al. 2012) integrated in the PhyloSuite (Zhang et al. 2020). The Markov chain Monte Carlo process was run 2,000,000 generations and trees were sampled every 100 generations with 2 × 4 chains. Stationarity was achieved when the average standard deviation of split frequencies remained < 0.01. The first 25% of runs were discarded as burn-in. The outcomes from MrBayes were summed up by PhyloSuite and the consensus trees were rooted using *Leersia* species as outgroups.

## Results

### Rice species and their phylogenetic relationships

**Fig. 1:**
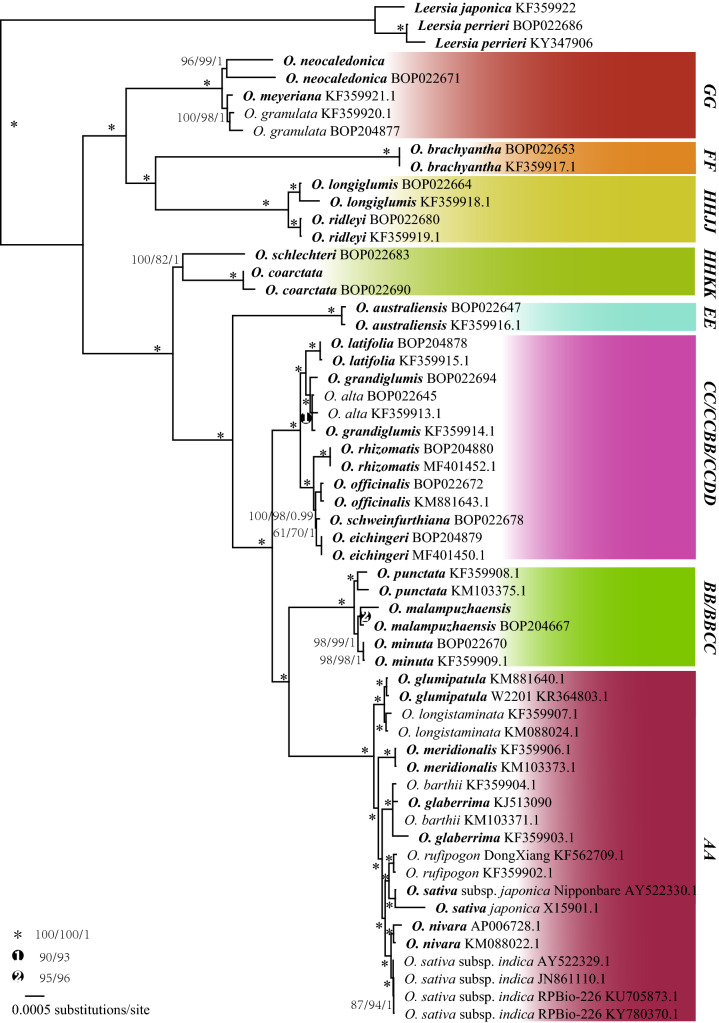
The maximum likelihood strict consensus tree based on the complete chloroplast genome sequences of all species in *Oryza*. The figures beside branches are bootstrap values of both maximum parsimony, maximum likelihood and Bayesian analyses. Genome types are given in bold capital letters on the right side

The phylogenetic relationships among *Oryza* species were reconstructed based on their complete chloroplast genomes, as well as the nuclear ITS, NP78, and R22 genes (Table S2). The eight clades in the complete chloroplast genome phylogeny matched exactly the eight genome types (Fig. 1). The species *O. malampuzhaensis* and *O. minuta* of the **BC** genome type formed a clade with *O. punctata*, indicating that a species of the **B** genome type was their maternal parent. *O. alta*, *O. grandiglumis*, and *O. latifolia* of the **CD** genome type and *O. schweinfurthiana* of the **BC** genome type formed a clade with species of the **C** genome type, suggesting their maternal parent belonged to the **C** genome type. Species with **HJ** and **HK** genome types did not form monophyletic clades, indicating that a species of the **H** genome type was their paternal parent.

**Fig. 2:**
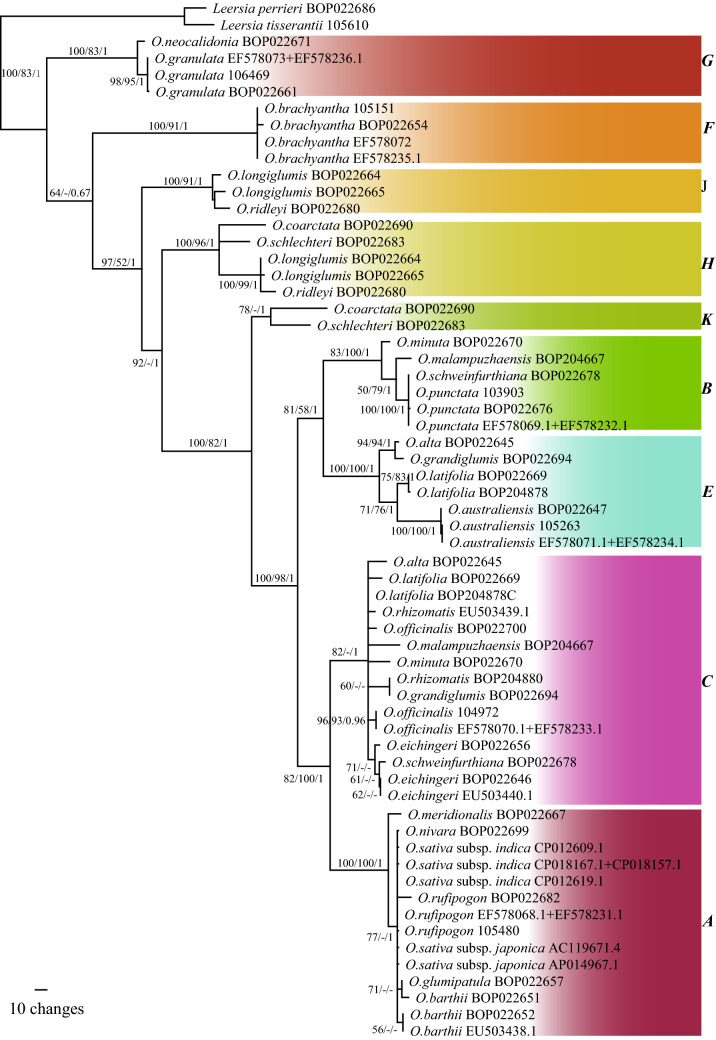
The maximum likelihood strict consensus tree based on concatenated sequences of nuclear NP78 and R22 genes of all species in *Oryza*. The figures beside branches are bootstrap values of both maximum parsimony, maximum likelihood and Bayesian analyses. Haplotypes are given in bold capital letters on the right side

Phylogeny based on the nuclear NP78 and R22 genes clarified the origins of allotetraploid species. Haplotypes of the same genome types formed monophyletic clades (Fig. 2). The clade comprising species with **F** and **G** genome types was located at the base, consistent with Fig. 1. The **H** haplotypes formed a clade independent of clades **J** and **K**, suggesting that a paternal parent with the **H** genome type had existed but then died out. The **D** haplotypes formed a clade with **E** haplotypes, indicating that the **D** genome type is a form of **E**. The species with a **BC** genome had independent origins, with *O. malampuzhaensis* = *O. officinalis* (**C**) × *O. punctata* (**B**) and *O. schweinfurthiana* = *O. punctata* (**B**) × *O. eichingeri* (**C**).

Genetic divergence between species of the same genome type was rather small, except between *O. schlechteri* and *O. coarctata*. No significant chloroplast genome divergence was observed between *O. alta* and *O. grandiglumis*, or between *O. barthii* and *O. glaberrima*. Minor divergence was detected between *O. glumipatula* and *O. longistaminata*. In contrast, chloroplast genome divergence was clearly noted between *O. sativa* subsp. *indica* and subsp. *japonica*. The former formed a monophyletic clade with *O. nivara*, and the latter formed a monophyletic clade with *O. rufipogon*.

### Rice-specific DNA barcodes

The hypervariable regions in the chloroplast genomes were identified by the sliding window method of DnaSP, and 36 regions (Table S4) were picked based on nucleotide diversity. Further evaluation of these 36 regions was carried out using the tree building method, and six high-resolution regions (*psaJ*-*rpl33*, *trnC-rpoB*, *rps16*-*trnQ*, *rpl22-rps19*, *trnK*-*matK*, and *ndhC-trnV*, Table 2) were finally chosen as rice-specific chloroplast DNA barcodes. While the above markers displayed higher nucleotide diversity and more variable sites than *rbcL*; overall, these two parameters were much higher in nuclear markers (Table 2).

**Table 2:** Primers designed to amplify six chloroplast regions and two nuclear genes

	Locus	Primer name	Primer sequence (5′–3′)	Nucleotide diversity	Fragment length	Variable site
1	*ndhC-trnV*	ndhC-f	ATCTGTTTTACCGAGAAGGTC	0.02012	1174–1232	325
		trnV-r	TATTCAGTTAAGACCATTCC			
2	*psaJ-rpl33*	psaJ-f	AATAGGTAGGGATGACAGG	0.01253	1115–1179	88
		rpl33-r	ATCGAACACAAGATGCTCC			
3	*rps16-trnQ*	rps16-f	TCGTGTCCTTCAAGTCGCACG	0.01212	1062–1214	105
		trnQ-r	ATAATACTGTTTATTAGTGTCGC			
4	*rpl22*	rpl22-f	TTGTTTGGAGGGGAAGTC	0.00835	1257–1363	83
		rps19-r	TGTAGCTCATCATTTATTGG			
5	*trnC-rpoB*	trnC-f	AAGCCTTGATTAATGAACC	0.01369	1242–1349	104
		rpoB-r	TAAGTATTTTATTGATCAGG			
6	*trnK-matK*	trnK-f	CTTGATCATTTATCAATCATTTC	0.01226	1523–1594	118
		matK-r	CACCCTGTTCTGACCATATTG			
7	NP78	NP78-1f	CGTCTGAAAAGCTTTTCTGGGAC	0.06379	1013–1066	358
		NP78-1r	TTATTATTGAAAACCAACTGAGC			
		NP78-2f	GCTCAGTTGGTTTTCAATAATAA			
		NP78-2r	AAAAAAAGTTAATTAAATGAG			
8	R22	R22-1f	ATAATAATTCAATAAATAG	0.11972	1106–1152	459
		R22-1r	GTTTGGTATCATTTGTGATATT			
		R22-2f	TCACACCTGGACAGAATATCAC			
		R22-2r	GTGTTGTTTTCATAAACAA			
9	*matK*			0.01243	1459	108
10	*rbcL*			0.00596	1428	43
11	ITS			0.04443	713	204
12	cpGenome			0.00573	141,850–145,469	

Information of nucleotide diversity and expected lengths, and number of variable sites of the eight markers together with three conventional DNA barcodes and the chloroplast genome superbarcode

### Discrimination powers of conventional, rice-specific, and super DNA barcodes

The different genome types within the *Oryza* genus have generally diverged sufficiently for most molecular markers to discriminate between them. The resolution of the various markers is tested by the presence of more than one species per genome type. Phylogenetic methods are the most reliable way to assign a sample to a species and the following comparisons were based on the maximum parsimonious phylogenies of nearly identical samples using different molecular markers, such as *matK*, *rbcL*, *psbA-trnH*, ITS, NP78 + R22, rice-specific barcodes, and the super barcode. Because of narrowly or incorrectly delimited species, molecular markers cannot discriminate between the following species pairs: *O. alta* and *O. grandiglumis* (Bao and Ge 2004), *O. barthii* and *O. glaberrima* (Wang et al. 2014), *O. glumipatula* and *O. longistaminata*, *O. granulata* and *O. meyeriana* (Gong et al. 2000), *O. minuta* and *O. malampuzhaensis*, *O. nivara* and *O. sativa* subsp. *indica*, and *O. sativa* subsp. *japonica* and *O. rufipogon*.

The *matK* gene had an aligned length of 1417 sites with 90 parsimony-informative characters when outgroups were included. This marker failed to discriminate between species of the **A**, **B**, and **C** genomes (Fig. S1).

The *rbcL* gene had an aligned length of 1428 sites with 50 parsimony-informative characters when outgroups were considered. This marker also failed to discriminate between species of the **A**, **B**, and **C** genomes (Fig. S2).

The *psbA-trnH* region had an aligned length of 515 sites with 10 parsimony-informative characters when outgroups and partial *rps19* were included. This marker could successfully identify only *O. brachyantha* and *O. sativa* subsp. *indica* (Fig. S3).

The nuclear ITS (including 5.8 s) had an aligned length of 713 sites with 162 parsimony-informative characters when outgroups were considered. The samples used for this marker differed slightly from those subjected to chloroplast markers because the sequences were difficult to amplify. Only one ITS copy was detected in several allotetraploid species. Phylogeny data based on ITS suggested that the **H** or **J** genome types originated from the **F** genome type (Fig. S4), a finding not supported by the other two nuclear genes. The ITS failed to discriminate between species of the **A** and **C** genome types.

The nuclear NP78 + R22 gene combination had an aligned length of 2218 sites with 722 parsimony-informative characters when outgroups were included. This marker combination failed to discriminate between species of the **A**, **B**, **C**, **H**, and **J** genome types (Fig. S5).

**Fig. 3:**
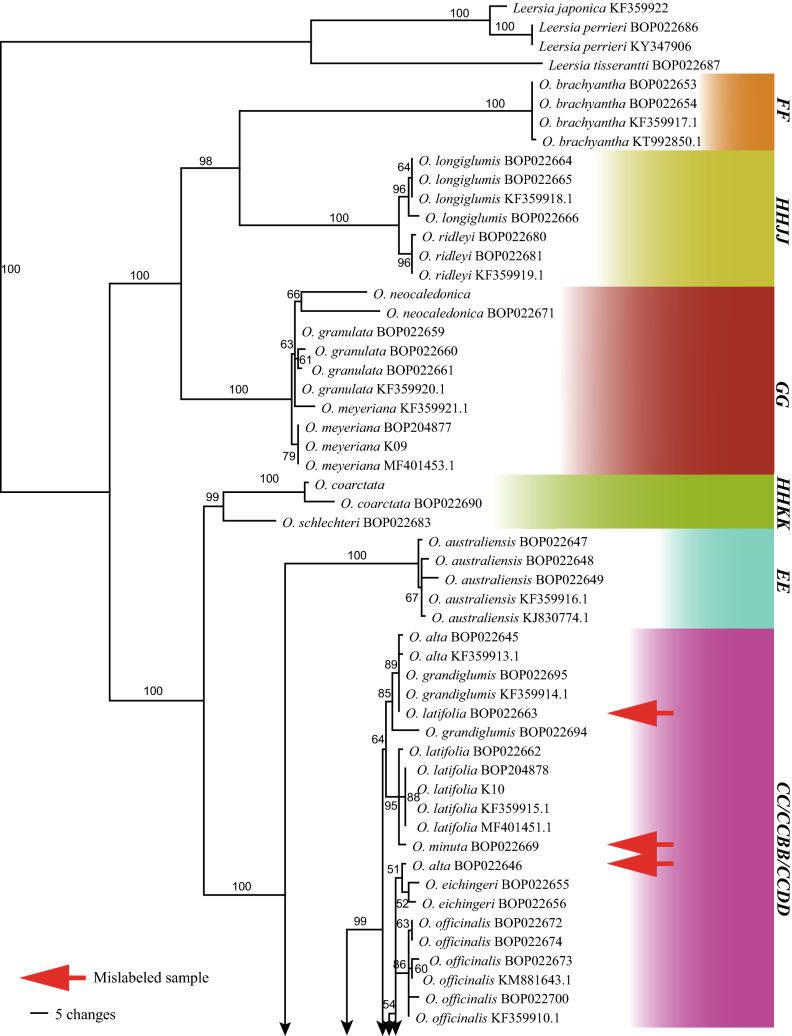

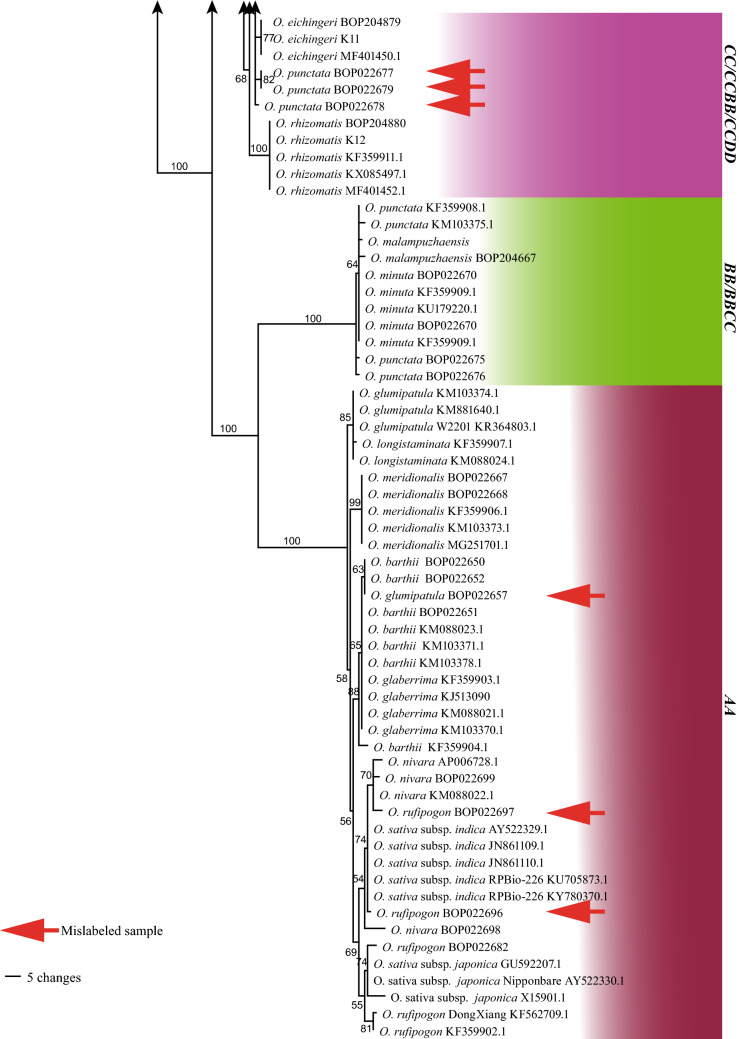
The maximum parsimony strict consensus tree based on the rice-specific chloroplast DNA barcode (concatenated six hypervariable regions) sequences of all species in *Oryza*, demonstrating the resolution of the marker, mislabeled samples and seeds identified. Accession numbers starting with “BOP” are seeds to be identified. The figures beside branches are bootstrap values and the genome types are given in bold capital letters on the right side

The rice-specific barcode consisted of six hypervariable chloroplast regions and had an aligned length of 7943 sites with 603 parsimony-informative characters when outgroups were considered. This marker combination resolved almost all species except *O. punctata* and *O. minuta* of the **B** genome type (Fig. 3).

Finally, the super DNA barcode of the complete chloroplast genome had an aligned length of 145,860 sites with 5048 parsimony-informative characters when outgroups were included. The super barcode exhibited the highest discriminating power, resolving all species using an insensitive but extremely reliable phylogenetic method (Fig. 1). Even though species of genome types **A** and **C** are very closely related and difficult to identify, the super barcode resolved them sufficiently well. Surprisingly, the species *O. rufipogon* + *O. sativa* subsp. *japonica* and *O. nivara* + *O. sativa* subsp. *indica* were separable using the super barcode.

### Identification of seeds and mislabeled samples from seed banks

Considering that the rice-specific barcode resolved almost all rice species, we used it to identify 53 accessions of seeds from seed banks or field collections. Nine (17%) mislabeled samples were found (Fig. 3). These samples were all from species-rich genome types **A** and **C**. This was not surprising, as in the **A** genome type, there is still some confusion between *O. rufipogon* and *O. nivara*, and between *O. glaberrima* (*O. barthii*) and *O. glumipatula*. Similarly, in the **C** genome type, there is confusion between diploid and tetraploid *O. punctata*, and among tetraploid *O. alta*, *O. latifolia*, and *O. minuta*.

## Discussion

### Species delimitation and taxonomy of rice

Correct species delimitation is a prerequisite for DNA barcoding. Although considerable efforts have been made on the taxonomy of *Oryza*, consensus has not been reached on the number of species in the genus and some controversies remain. So far, the phylogeny of all species is incomplete. Phylogeny based on the chloroplast genome (Fig. 1) indicates that species of the **E** (*O. australiensis*) and **F** (*O. brachyantha*) genome types are monospecific and relatively isolated from other species. Species pairs have been found between *O. meyeriana* and *O. neocaledonica* of the **G** genome type, between *O. longiglumis* and *O. ridleyi* of the **HJ** genome type, and between *O. coarctata* of the **KL** genome type and *O. schlechteri* of the **HK** genome type (Lu and Ge 2003). Phylogeny based on the nuclear N78 + R22 marker (Fig. 2) revealed that the **L** genome did not exist, while *O. coarctata* belonged to the **HK** rather than the **KL** genome type. Species belonging to the **HJ** and **HK** genome types share a common paternal progenitor with the **H** genome, a now-extinct species originating somewhere in Irian Jaya, Indonesia or Papua New Guinea.

Major identification problems exist among species of the **A**, **B**, and **C** genome types. As with the **H** genome type, the **D** genome type is found only in South and Central American species, such as *O. alta*, *O. latifolia*, and *O. grandiglumis*, with **CCDD** genomes. Interestingly, the **D** genome type isolated from the sample BOP022669 was identified as *O. latifolia* and formed a clade with *O. australiensis* of the **E** genome type (Fig. 2). Phylogeny indicates that the **D** genome type is very likely a variant of the **E** genome type, if not **E** itself, confirming earlier results (Bao and Ge 2004; Ge et al. 1999).

There is a general correlation between molecular divergence and species delimitation (Lefébure et al. 2006). Little chloroplast genome divergence was observed between *O. alta* and *O. grandiglumis* and their conspecific nature was suggested (Bao and Ge 2004) based on nuclear genes. Considering the trivial morphological difference between *O. alta* and *O. grandiglumis*, the former becomes often a synonym of the latter instead of *O. latifolia* Desv., as for example on “The Plant List” (http://www.theplantlist.org/tpl1.1/record/kew-426597).

Within the **BC** genome type, the two Asian species *O. malampuzhaensis* and *O. minuta* originated by hybridization between *O. punctata* as maternal parent and *O. officinalis* as paternal parent (Zou et al. 2015). In contrast, for the African species *O. schweinfurthiana*, *O. eichingeri* served as maternal parent and *O. punctata* as paternal parent. Considering insignificant morphological differences between *O. malampuzhaensis* and *O. minuta*, the former could be regarded as a synonym of the latter. Given that *O. schweinfurthiana* is an allotetraploid with a different maternal parent compared to *O. minuta*, it should be considered a distinct species instead of merging it within *O. punctata*.

Misidentification of plant material is very common within the **A** genome type due to incorrect discrimination between species. All these species diverged within a short period by a radiation event (Wambugu et al. 2015; Zhang et al. 2014). Some species pairs exhibit neither obvious morphological difference nor remarkable genetic divergence. A first instance of confusion involves the African cultivated rice *O. glaberrima* and its wild progenitor *O. barthii*. No obvious genetic divergence has happened between their chloroplast genomes, which confirms similar results based on nuclear genes (Li et al. 2011; Wang et al. 2014). They often grow side by side in the field without ecological niche differentiation. Hence, *O*. *barthii* should be considered a synonym of *O. glaberrima* or a wild type.

A second confusing case involves the Asian cultivated rice *O. sativa* and its wild progenitors *O. nivara* and *O. rufipogon*. The Asian cultivated rice was divided into two subspecies, subsp. *indica* and subsp. *japonica*, in spite of naked names. Although the two subspecies are reproductively isolated, differ significantly in morphology and physiology, and were domesticated separately in the Himalayan mountain range and southern China (Londo et al. 2006), their taxonomic status has never been questioned. Our molecular phylogenies and almost all previous studies such as that by Wambugu et al. (2015) have confirmed that the two cultivated subspecies have the closest wild species of their own. It is very clear now that *O. sativa* subsp. *indica* is domesticated from *O. nivara* and that *O. sativa* subsp. *japonica* comes from *O. rufipogon*. Because the type of *O. sativa* belongs to *O. sativa* subsp. *japonica*, *O. sativa* must be retained in this cultivated subspecies with an autonomous name. Therefore, the two subspecies should be detached and renamed as *O. sativa* subsp. *sativa* (syn. *O. sativa* subsp. *japonica*) and *O. nivara* subsp. *indica* (syn. *O. sativa* subsp. *indica*). The names of their wild progenitors, *O. nivara* and *O. rufipogon*, have to be changed accordingly to *O. sativa* subsp. *rufipogon* (syn. *O. rufipogon*) and *O. nivara* subsp. *nivara* (syn. *O. nivara*). In 1970, a male sterile interspecific hybrid between *O. nivara* subsp. *indica* (= *O. sativa* subsp. *indica*) and *O. sativa* subsp. *sativa* (= *O. sativa* subsp. *japonica*) was discovered at a farm in Hainan province, China. The reproductive isolation between these subspecies was broken artificially and partially fertile F1 hybrid rice was used to produce fertile F2 hybrids as a new cultivar, which exhibited considerable hybrid vigor. Subsequent hybridization, however, created taxonomic problems regarding the correct identification of the two kinds of rice and their wild progenitors, resulting in many incorrectly labeled sequences being deposited in GenBank.

After synonymizing *O. longistaminata* under *O. glumipatula* and including *Porteresia coarctata* (Roxb.) Tateoka into *Oryza* (= *O. coarctata* Roxb.), 21 species are now recognized in the *Oryza* genus (supporting text S1).

### Conventional DNA barcodes of rice

Three chloroplast regions (*matK*, *psbA-trnH*, and *rbcL*) and one nuclear region (ITS) represent conventional DNA barcodes for higher plants (Hollingsworth et al. 2009; Kress et al. 2005). Chloroplast regions perform differently in different plant groups. Here, we extracted these regions and conducted phylogenetic analyses to evaluate their suitability for species resolution. Their performance was barely satisfactory in *Oryza.* Generally, the *matK* gene offers higher resolution than *rbcL*, but in *Oryza*, it did not perform much better. Fewer than half of the 21 species were reliably (bootstrap values > 75%) resolvable. Both barcodes failed to discriminate between species of the **A**, **B**, and **C** genome types. Moreover, a combination of *matK* + *rbcL* did not improve the situation, because both barcodes resolved almost the same species without complementation. The *psbA-trnH* intergenic spacer, one of the most variable regions in chloroplast genomes, performed similarly poorly with only one identifiable species. This is probably due to the insertion of *rps19*, which replaced the spacer with *rps19* sequences.

The nuclear ITS afforded similar resolution as conventional chloroplast regions. Although there are 10 allotetraploid species in *Oryza*, only one genome was detected in *O. coarctata* (**KL**), *O. ridleyi* (**HJ**), and *O. schlechteri* (**HK**). However, two kinds of sequences were observed in *O. longiglumis* (**HJ**), one of them was similar to that of *O. ridleyi*, and the other was similar to that of *O. brachyantha*, a phenomenon never reported previously. Similarly, only the **C** genome type was confirmed in *O. alta* and *O. grandiglumis*, whereas the **B** genome type defined *O. malampuzhaensis*. Both **B** and **C** genome types were detected in *O. schweinfurthiana*. The sequences deposited in GenBank include only one kind of sequence for species of the **BC** and **CD** genome types, which is probably because of concerted evolution of the ITS in relatively old tetraploids. Only newly formed tetraploids such as *O. schweinfurthiana* maintain both **B** and **C** genome types.

### Rice-specific DNA barcodes

Most species in the *Oryza* genus have an evolutionary history of only a few million years. Very limited genetic variation has accumulated within such a short time and conventional DNA barcodes do not work well at species level, especially for those belonging to the **A**, **B**, and **C** genome types. The two most variable genes (NP78 and R22) picked out from 142 nuclear genes tested by Zou et al. (2008) served here as rice-specific nuclear DNA barcodes. Despite sequencing difficulties arising from multiple copies in tetraploid species, the combined marker performed sufficiently well. It is unlikely for the two genes to have diverged significantly in different species, thus explaining why they could discriminate between species of the same genome types.

Although species of the **A**, **B** or **C** genome types are very closely related, complete chloroplast genomes have accumulated enough variations to discriminate between them and all rice species are identifiable even with phylogenetic methods. Owing to the single-copy nature of chloroplast genes, mutations in chloroplast genomes become fixed and spread more quickly than those in nuclear genomes. Such mutations may not reflect a true phylogeny but are adequate for species discrimination.

The powerful performance of the complete chloroplast genome for species identification does not imply that it should be used in routine plant material identifications. There are some sensible shortcuts one can take, as a very large proportion of the chloroplast genome does not contribute much to species discrimination. The most variable regions could be an epitome of the whole genome. Here, six hypervariable regions in the chloroplast genome were selected and their combination served as rice-specific DNA barcodes. This epitome worked almost as well as the entire genome in terms of species discrimination using rice seeds from seed banks or field collections.

### Identification of rice seeds

Although some seed morphological characteristics can be used successfully for seed identification, it is very difficult even for taxonomists to apply them correctly and there are species whose seeds are difficult to identify by morphology only. This explains why the wrong seeds were occasionally distributed to users. Here, we show that 17% of seeds were mislabeled, a figure high enough to deserve serious consideration. Although no algorithm has improved the assignment of specimens to species (Spouge and Mariño-Ramírez 2012), our findings suggest that phylogenetic methods offer the most reliable but also the least sensitive approach in this respect. At species level, samples in a monophyletic clade with a reasonable bootstrap support belong to the same species.

## Electronic supplementary material

Below is the link to the electronic supplementary material.

Figure S1. The maximum parsimony strict consensus tree based on the conventional DNA barcode *matK* sequences of all species in *Oryza*, demonstrating the resolution of the marker. The figures beside branches are bootstrap values (PDF 149.8 kb)

Figure S2. The maximum parsimony strict consensus tree based on the conventional DNA barcode *rbcL* sequences of all species in *Oryza*, demonstrating the resolution of the marker. The figures beside branches are bootstrap values 2 (PDF 145.6 kb)

Figure S3. The maximum parsimony strict consensus tree based on the conventional DNA barcode *psbA-trnH* sequences of all species in *Oryza*, demonstrating the resolution of the marker. The figures beside branches are bootstrap values (PDF 137.2 kb)

Figure S4. The maximum parsimony strict consensus tree based on the conventional DNA barcode ITS sequences of all species in *Oryza*, demonstrating the resolution of the marker. The figures beside branches are bootstrap values (PDF 164.5 kb)

Figure S5. The maximum parsimony strict consensus tree based on the rice-specific nuclear DNA barcode of NP78 + R22 sequences of all species in *Oryza*, demonstrating the resolution of the marker. The figures beside branches are bootstrap values (PDF 41.4 kb)

Supplementary material 6 (DOCX 15.0 kb)

Supplementary material 7 (DOCX 16.9 kb)

Supplementary material 8 (DOCX 15.8 kb)

Supplementary material 9 (DOCX 19.1 kb)

Supplementary material 10 (DOCX 17.6 kb)
