# Supplementary figures and images for: DNA barcoding of Oryza: conventional, specific, and super barcodes
Source: Plant Mol Biol. 2020 Sep 3;105(3):215–28. doi: 10.1007/s11103-020-01054-3 (PMC7858216; doi:10.1007/s11103-020-01054-3)

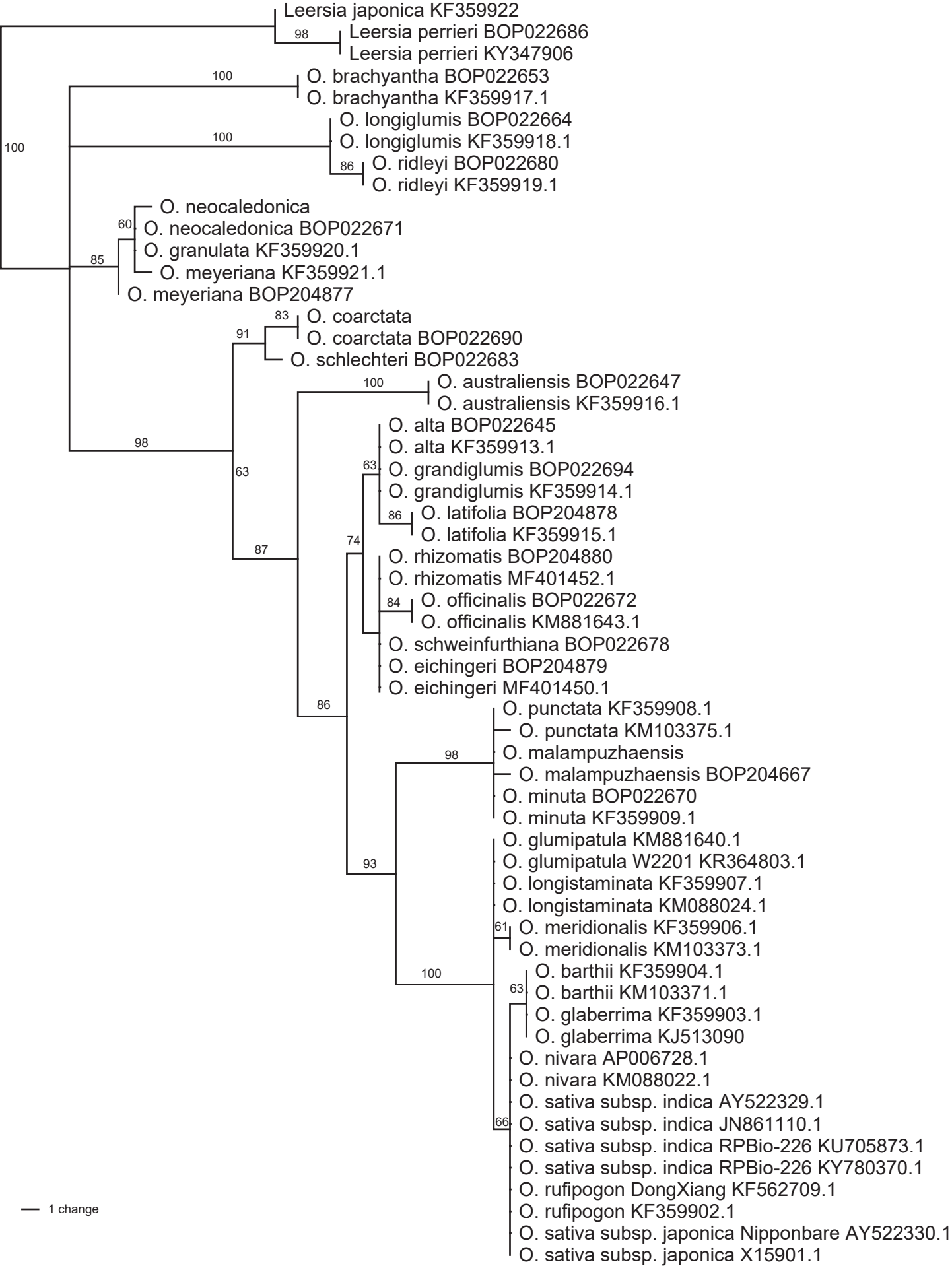

Supplement: Supplementary file 1 — Figure S1. The maximum parsimony strict consensus tree based on the conventional DNA barcode matK sequences of all species in Oryza, demonstrating the resolution of the marker. The figures beside branches are bootstrap values (PDF 149.8 kb) [file 11103_2020_1054_MOESM1_ESM.pdf]

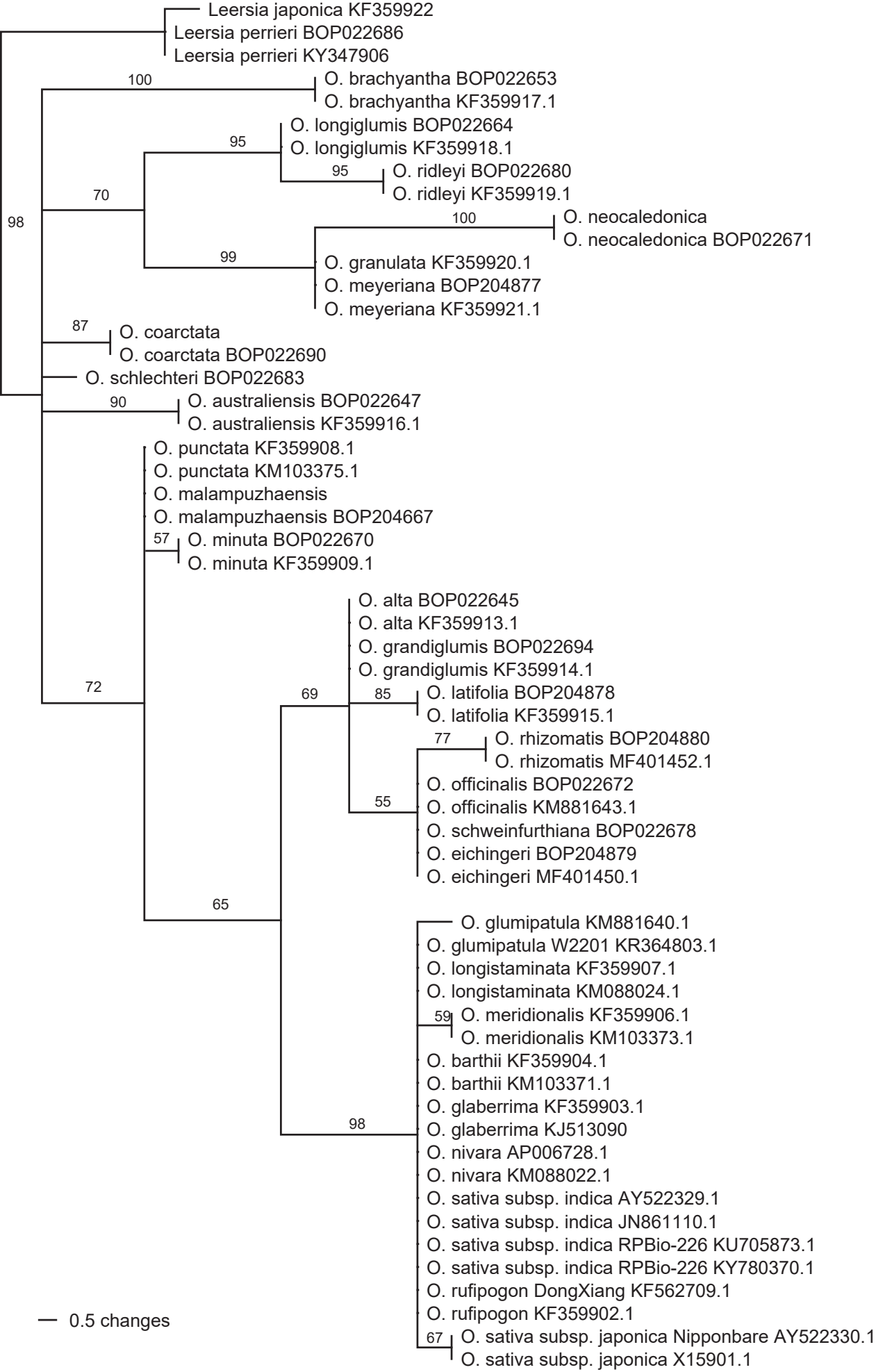

Supplement: Supplementary file 2 — Figure S2. The maximum parsimony strict consensus tree based on the conventional DNA barcode rbcL sequences of all species in Oryza, demonstrating the resolution of the marker. The figures beside branches are bootstrap values 2 (PDF 145.6 kb) [file 11103_2020_1054_MOESM2_ESM.pdf]

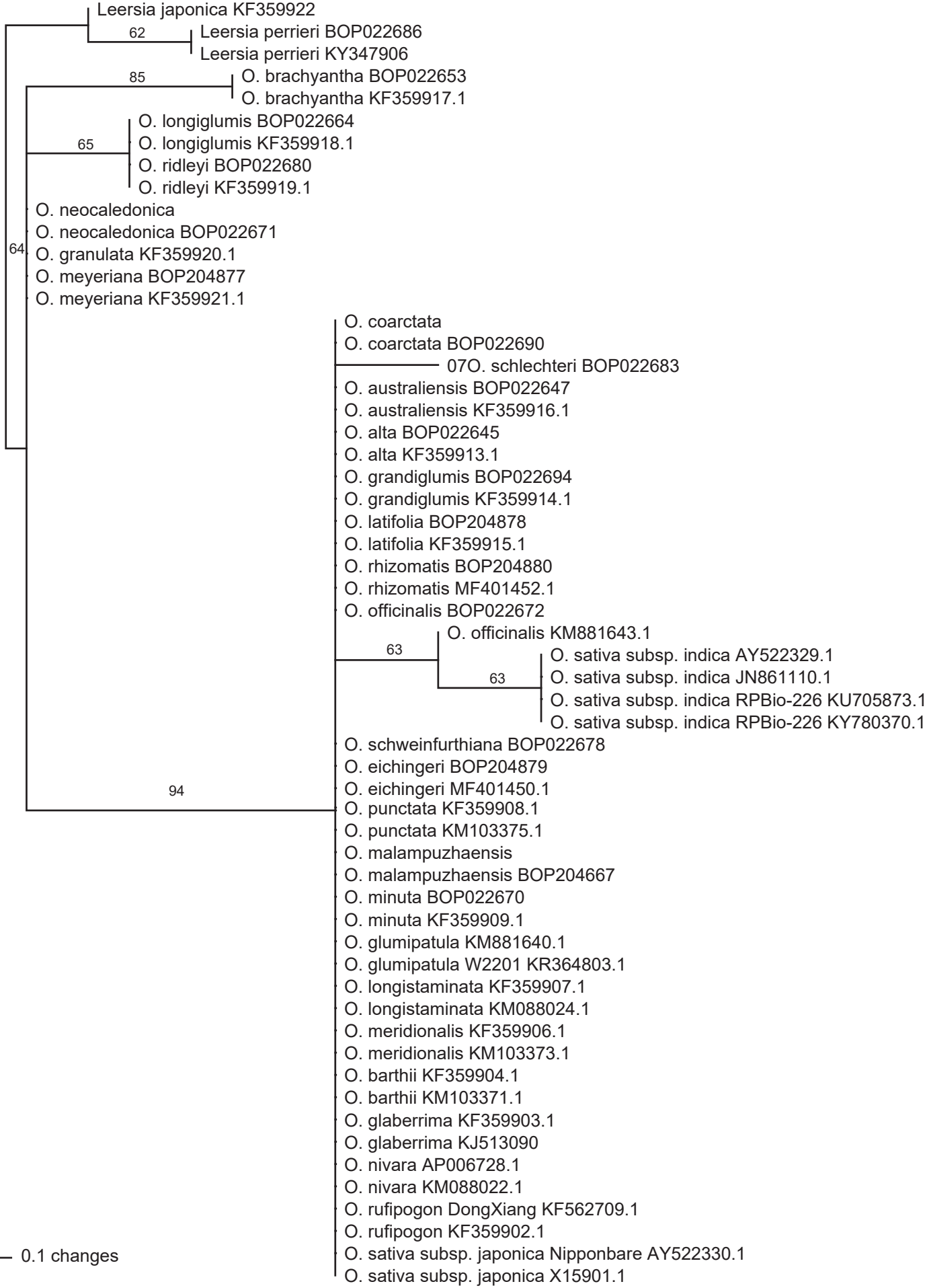

Supplement: Supplementary file 3 — Figure S3. The maximum parsimony strict consensus tree based on the conventional DNA barcode psbA-trnH sequences of all species in Oryza, demonstrating the resolution of the marker. The figures beside branches are bootstrap values (PDF 137.2 kb) [file 11103_2020_1054_MOESM3_ESM.pdf]

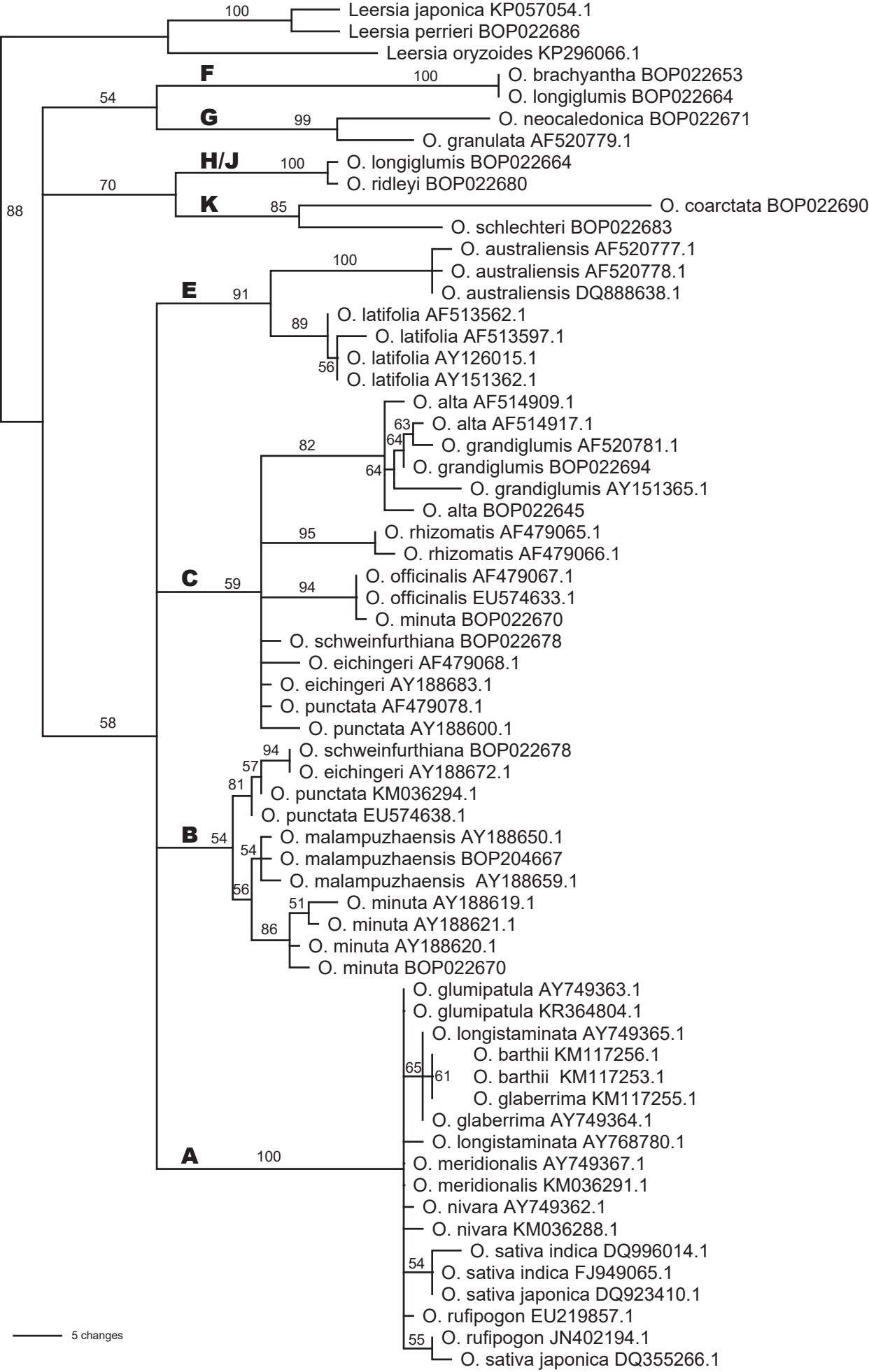

Supplement: Supplementary file 4 — Figure S4. The maximum parsimony strict consensus tree based on the conventional DNA barcode ITS sequences of all species in Oryza, demonstrating the resolution of the marker. The figures beside branches are bootstrap values (PDF 164.5 kb) [file 11103_2020_1054_MOESM4_ESM.pdf]

## Bootstrap consensus tree

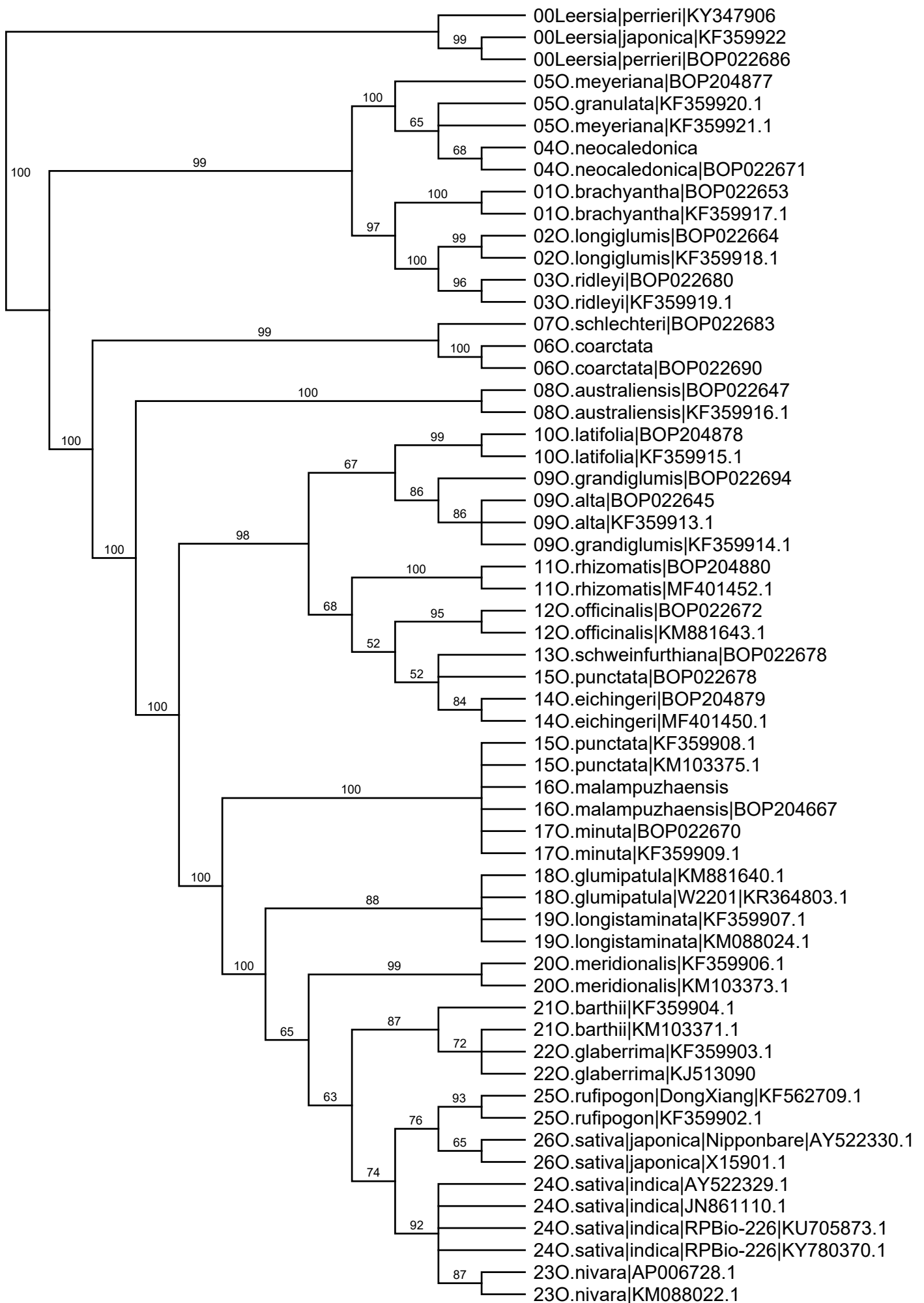

Supplement: Supplementary file 5 — Figure S5. The maximum parsimony strict consensus tree based on the rice-specific nuclear DNA barcode of NP78 + R22 sequences of all species in Oryza, demonstrating the resolution of the marker. The figures beside branches are bootstrap values (PDF 41.4 kb) [file 11103_2020_1054_MOESM5_ESM.pdf]
