# Supplementary material for: DNA barcoding of Oryza: conventional, specific, and super barcodes
Source: Plant Mol Biol. 2020 Sep 3;105(3):215–28. doi: 10.1007/s11103-020-01054-3 (PMC7858216; doi:10.1007/s11103-020-01054-3)
Supplement: Supplementary file 6 — Supplementary material 6 (DOCX 15.0 kb) [file 11103_2020_1054_MOESM6_ESM.docx]

Table S1. Generally accepted species in *Oryza* with chromosome number, genome type and distribution information.

|  | **Taxon** | **2n=** | Genome | **Distribution** |
| --- | --- | --- | --- | --- |
| 1 | *O. alta* Swallen | 48 | *CCEE* (CCDD) | South and Central America |
| 2 | *O. australiensis* Domin. | 24 | *EE* | Tropical Australia |
| 3 | *O. barthii* A. Chev. | 24 | *AA* | Africa |
| 4 | *O. brachyantha* Chev. et Roehr. | 24 | *FF* | Africa |
| 5 | *O. coarctata* Roxb. | *48* | *HHKK* | India, Sri Lanka, Bangladesh, and Myanmar |
| 6 | *O. eichingeri* Peter | 24, 48 | *CC* | South Asia and East Africa |
| 7 | *O. glaberrima* Steud. | 24 | *AA* | West Africa |
| 8 | *O. glumipatula* Steud. | 24 | *AA* | South and Central America |
| 9 | *O. grandiglumis* (Doell) Prod. | 48 | *CCEE* (CCDD) | South and Central America |
| 10 | *O. latifolia* Desv. | 48 | *CCEE* (CCDD) | South and Central America |
| 11 | *O. longiglumis* Jansen | 48 | *HHJJ* | Irian Jaya, Indonesia and Papua New Guinea |
| 12 | *O. longistaminata* A. Chev. et Roehr. | 24 | *AA* | Africa |
| 13 | *O. malampuzhaensis* Kishn.et Chandr. | 48 | *BBCC* | India |
| 14 | *O. meridionalis* Ng | 24 | *AA* | Tropical Australia |
| 15 | *O. meyeriana* (Zoll. & Moritzi) Baill. | 24 | *GG* | Asia |
| 16 | *O. minuta* J. S. Presl. et C. B. Presl. | 48 | *BBCC* | Philippine and Papua New Guinea |
| 17 | *O. neocaledonica* Morat | 24 | *GG* | New Caledonia |
| 18 | *O. nivara* Sharma et Shastry | 24 | *AA* | Tropical and subtropical Asia |
| 19 | *O. officinalis* Wall ex Watt | 24, 48 | *CC* | Tropical and subtropical Asia, Tropical Australia |
| 20 | *O. punctata* Kotschy ex Steud. | 24 | *BB* | Africa |
| 21 | *O. rhizomatis* Vaughan | 24 | *CC* | Sir Lanka |
| 22 | *O. ridleyi* Hook f. | 48 | *HHJJ* | South Asia |
| 23 | *O. rufipogon* Griff. | 24 | *AA* | Tropical and subtropical Asia, Tropical Australia |
| 24 | *O. sativa* L. subsp. *indica* S. Kato | 24 | *AA* | Cultivated world wide |
| 25 | *O. sativa* L. subsp. *japonica* S. Kato | 24 | *AA* | Cultivated world wide |
| 26 | *O. schlechteri* Pilger | 48 | *HHKK* | Papua New Guinea and Irian Jaya, Indonesia |
| 27 | *O. schweinfurthiana* Prod. | 48 | *CCBB* | Africa |
