# Supplementary material for: DNA barcoding of Oryza: conventional, specific, and super barcodes
Source: Plant Mol Biol. 2020 Sep 3;105(3):215–28. doi: 10.1007/s11103-020-01054-3 (PMC7858216; doi:10.1007/s11103-020-01054-3)
Supplement: Supplementary file 7 — Supplementary material 7 (DOCX 16.9 kb) [file 11103_2020_1054_MOESM7_ESM.docx]

Table S2. Sequences generated in this study and sequence accession number. Characters in brackets represent different genome types.

| Species | Voucher | Chloroplast genome | ITS | NP78 | R22 |
| --- | --- | --- | --- | --- | --- |
| *Leersia perrieri* | BOP022686 | MT726934 | MT676802 | MT682015 | MT682051 |
| *Oryza alta* | BOP022645 | MT726934 | MT676791 | MT681989(C)/MT681990(E) | MT682024(C)/MT682025(E) |
| *Oryza australiensis* | BOP022647 | MT726929 |  | MT681992 | MT682027 |
| *Oryza barthii* | BOP022651 |  |  | MT681993 | MT682028 |
| *Oryza barthii* | BOP022652 |  |  | MT681994 | MT682029 |
| *Oryza brachyantha* | BOP022653 | MT726938 | MT676792 |  |  |
| *Oryza brachyantha* | BOP022654 |  |  | MT681995 | MT682030 |
| *Oryza coarctata* | BOP022690 | MT726929 | MT676803 | MT682016(H)/MT682017(K) | MT682052(H)/MT682053(K) |
| *Oryza eichingeri* | BOP022646 |  |  | MT681991 | MT682026 |
| *Oryza eichingeri* | BOP022656 |  |  | MT681996 | MT682031 |
| *Oryza eichingeri* | BOP204879 | MF401450 |  |  |  |
| *Oryza glumipatula* | BOP022657 |  |  | MT681997 |  |
| *Oryza grandiglumis* | BOP022694 | MT726928 | MT676804 | MT682018(C)/MT682019(E) | MT682054(C)/MT682055(E) |
| *Oryza granulata* | BOP022661 |  |  | MT681998 | MT682032 |
| *Oryza granulata* | BOP204877 | MF401453 |  |  |  |
| *Oryza latifolia* | BOP022669 |  |  | MT682002(C)/MT682003(E) | MT682038(C)/MT682039(E) |
| *Oryza latifolia* | BOP204878 | MF401451 |  |  | MT682060(C)/MT682061(E) |
| *Oryza longiglumis* | BOP022664 | MT726933 | MT676793(H)/MT676794(J) |  | MT682033(H)/MT682034(J) |
| *Oryza longiglumis* | BOP022665 |  |  | MT681999(H)/MT682000(J) | MT682035(H)/MT682036(J) |
| *Oryza malampuzhaensis* | BOP204667 | MT726934 | MT676805 | MT682022(B)/MT682023(C) | MT682058(B)/MT682059(C) |
| *Oryza meridionalis* | BOP022667 |  |  | MT682001 | MT682037 |
| *Oryza minuta* | BOP022670 | MT726938 | MT676795(B)/MT676796(C) | MT682004(B)/MT682005(C) | MT682040(B)/MT682041(C) |
| *Oryza neocalidonia* | BOP022671 | MT726926 | MT676797 | MT682006 | MT682042 |
| *Oryza nivara* | BOP022699 |  |  | MT682020 | MT682056 |
| *Oryza officinalis* | BOP022672 | MT726929 | MT676798(B)/MT676799(C) | MT682008(B)/MT682009(C) |  |
| *Oryza officinalis* | BOP022700 |  |  | MT682021 | MT682057 |
| *Oryza punctata* | BOP022676 |  |  | MT682007 | MT682043 |
| *Oryza rhizomatis* | BOP204880 | MF401452 |  |  | MT682062 |
| *Oryza ridleyi* | BOP022680 | MT726934 | MT676800 | MT682010(H)/MT682011(J) | MT682047(H)/MT682048(J) |
| *Oryza rufipogon* | BOP022682 |  |  | MT682012 |  |
| *Oryza schlechteri* | BOP022683 | MT726927 | MT676801 | MT682013(H)/MT682014(K) | MT682049(H)/MT682050(K) |
| *Oryza schweinfurthiana* | BOP022678 | MT726932 |  |  | MT682044(B)/MT682045(C) |
