# Supplementary material for: DNA barcoding of Oryza: conventional, specific, and super barcodes
Source: Plant Mol Biol. 2020 Sep 3;105(3):215–28. doi: 10.1007/s11103-020-01054-3 (PMC7858216; doi:10.1007/s11103-020-01054-3)
Supplement: Supplementary file 8 — Supplementary material 8 (DOCX 15.8 kb) [file 11103_2020_1054_MOESM8_ESM.docx]

Table S3. Chloroplast genomes of *Oryza* and *Leersia* species downloaded from GenBank.

| Genus | Species | GenBank # | Total length |
| --- | --- | --- | --- |
| *Oryza* | *alta* | KF359913.1 | 143446 (8503 indels) |
| *Oryza* | *australiensis* | KF359916.1 | 143446 (8456 indels) |
| *Oryza* | *barthii* | KF359904.1 | 143446 (9097 indels) |
| *Oryza* | *barthii* | KM103371.1 | 143446 (9080 indels) |
| *Oryza* | *brachyantha* | KF359917.1 | 143446 (9034 indels) |
| *Oryza* | *coarctata* | * | 28550 (6873 indels) |
| *Oryza* | *eichingeri* | MF401450.1 | 143446 (8861 indels) |
| *Oryza* | *glaberrima* | KF359903.1 | 143446 (9017 indels) |
| *Oryza* | *glaberrima* | KJ513090 | 143446 (11455 indels) |
| *Oryza* | *glumipatula* | KM881640.1 | 143446 (9095 indels) |
| *Oryza* | *glumipatula* | KR364803.1 | 143446 (9103 indels) |
| *Oryza* | *grandiglumis* | KF359914.1 | 143446 (8528 indels) |
| *Oryza* | *granulata* | KF359920.1 | 143446 (7736 indels) |
| *Oryza* | *latifolia* | KF359915.1 | 143446 (8488 indels) |
| *Oryza* | *longiglumis* | KF359918.1 | 141850 (8034 indels) |
| *Oryza* | *longistaminata* | KF359907.1 | 143446 (9121 indels) |
| *Oryza* | *longistaminata* | KM088024.1 | 143446 (9120 indels) |
| *Oryza* | *malampuzhaensis* | * | 30308 (7130 indels) |
| *Oryza* | *meridionalis* | KF359906.1 | 143446 (9125 indels) |
| *Oryza* | *meridionalis* | KM103373.1 | 143446 (9122 indels) |
| *Oryza* | *meyeriana* | KF359921.1 | 143446 (7545 indels) |
| *Oryza* | *minuta* | KF359909.1 | 143446 (9083 indels) |
| *Oryza* | *neocaledonica* | * | 30295 (8282 indels) |
| *Oryza* | *nivara* | AP006728.1 | 144973 (9634 indels) |
| *Oryza* | *nivara* | KM088022.1 | 143446 (9162 indels) |
| *Oryza* | *officinalis* | KM881643.1 | 144973 (10120 indels) |
| *Oryza* | *punctata* | KF359908.1 | 143446 (9098 indels) |
| *Oryza* | *punctata* | KM103375.1 | 143446 (9075 indels) |
| *Oryza* | *rhizomatis* | MF401452.1 | 143445 (8930 indels) |
| *Oryza* | *ridleyi* | KF359919.1 | 141850 (7943 indels) |
| *Oryza* | *rufipogon* | KF359902.1 | 143446 (9091 indels) |
| *Oryza* | *rufipogon* | KF562709.1 | 143446 (9141 indels) |
| *Oryza* | *sativa* subsp. *indica* | AY522329.1 | 141657 (9182 indels) |
| *Oryza* | *sativa* subsp. *indica* | JN861110.1 | 143446 (9225 indels) |
| *Oryza* | *sativa* subsp. *indica* | KU705873.1 | 143446 (9185 indels) |
| *Oryza* | *sativa* subsp. *indica* | KY780370.1 | 143446 (9188 indels) |
| *Oryza* | *sativa* subsp. *japonica* | AY522330.1 | 143446 (9127 indels) |
| *Oryza* | *sativa* subsp. *japonica* | X15901.1 | 143446 (9155 indels) |
| *Leersia* | *japonica* | KF359922 | 143446 (9609 indels) |
| *Leersia* | *perrieri* | KY347906 | 143446 (7482 indels) |
| * | cancatenated fragments | |  |
