# Supplementary material for: DNA barcoding of Oryza: conventional, specific, and super barcodes
Source: Plant Mol Biol. 2020 Sep 3;105(3):215–28. doi: 10.1007/s11103-020-01054-3 (PMC7858216; doi:10.1007/s11103-020-01054-3)
Supplement: Supplementary file 9 — Supplementary material 9 (DOCX 19.1 kb) [file 11103_2020_1054_MOESM9_ESM.docx]

Table S4. Initial variability evaluation of the chloroplast genomes of *Oryza* species and 36 variable regions were listed. S: variable sites; h: number of haplotypes; Pi: nucleotide diversity; k: average number of nucleotide differences.

| 0 | Region | Gene | Sequence | Aligned length | Used length | S | h | Pi | k |
| --- | --- | --- | --- | --- | --- | --- | --- | --- | --- |
| 1 | 001702-003259 | *matK* | 36 | 1564 | 1101 | 78 | 25 | 0.01174 | 12.93 |
| 2 | 004566-005472 | *rps16* | 29 | 933 | 565 | 31 | 15 | 0.00824 | 4.658 |
| 3 | 005571-006663 | *rps16-trnQ* | 15 | 1104 | 460 | 43 | 14 | 0.02253 | 10.362 |
| 4 | 007190-007618 | *psbK-I* | 16 | 366 | 261 | 3 | 3 | 0.00227 | 0.592 |
| 5 | 007939-008630 | *trnS* | 32 | 715 | 281 | 13 | 9 | 0.00621 | 2.587 |
| 6 | 011631-012858 | *trnS-trnfM* | 46 | 1277 | 331 | 26 | 18 | 0.01304 | 4.318 |
| 7 | 012954-013669 | *trnG-trnfM* | 15 | 739 | 715 | 28 | 11 | 0.01023 | 7.314 |
| 8 | 015155-016199 | *trnT-trnD* | 15 | 1115 | 626 | 40 | 13 | 0.01623 | 10.162 |
| 9 | 018125-019234 | *trnC-rpoB* | 14 | 1146 | 1039 | 59 | 12 | 0.01502 | 15.604 |
| 10 | 020943-021385 | *rpoB* | 30 | 459 | 351 | 3 | 4 | 0.00155 | 0.545 |
| 11 | 023505-024019 | *rpoC* | 32 | 529 | 396 | 3 | 4 | 0.00089 | 0.351 |
| 12 | 027535-029268 | *rpoc2* | 24 | 1419 | 1419 | 3 | 3 | 0.00097 | 1.38 |
| 13 | 031137-032014 | *atpI-atpH* | 12 | 923 | 800 | 47 | 11 | 0.01475 | 11.803 |
| 14 | 032267-032831 | *atp8-atpF* | 14 | 564 | 375 | 2 | 2 | 0.00264 | 0.989 |
| 15 | 032856-033735 | *AtpF* | 15 | 893 | 862 | 27 | 9 | 0.0075 | 6.467 |
| 16 | 041104-041990 | *psaA* | 14 | 899 | 876 | 18 | 9 | 0.00435 | 3.813 |
| 17 | 042900-044077 | *ycf3* | 32 | 1177 | 52 | 1 | 2 | 0.00931 | 0.484 |
| 18 | 045781-047548 | *trnT-trnL* | 53 | 1521 | 417 | 66 | 25 | 0.01295 | 5.399 |
| 19 | 048027-048420 | *ndhJ* | 13 | 395 | 313 | 1 | 2 | 0.00049 | 0.154 |
| 20 | 049362-050428 | *trnV-ndhC* | 54 | 1098 | 340 | 48 | 21 | 0.01781 | 6.055 |
| 21 | 050457-051219 | *trnM-trnV* | 15 | 774 | 728 | 32 | 11 | 0.01031 | 7.505 |
| 22 | 053104-054093 | *atpB-rbcL* | 69 | 1041 | 351 | 31 | 28 | 0.00757 | 2.658 |
| 23 | 056536-057172 | *rbcL* | 38 | 657 | 83 | 1 | 2 | 0.0018 | 0.149 |
| 24 | 056536-057222 | *rbcL-psaI* | 9 | 694 | 645 | 26 | 7 | 0.01258 | 8.111 |
| 25 | 060487-061961 | *petA-psbL* | 17 | 1489 | 1384 | 45 | 12 | 0.00669 | 9.265 |
| 26 | 064304-065384 | *psaJ-rpl33* | 23 | 1102 | 607 | 40 | 14 | 0.01574 | 9.553 |
| 27 | 066468-068213 | *clpP-rps12* | 16 | 1770 | 890 | 40 | 9 | 0.00982 | 8.742 |
| 28 | 070947-072073 | *psbH-petB* | 15 | 1143 | 1120 | 44 | 13 | 0.00954 | 10.686 |
| 29 | 074014-075284 | *rpoA* | 11 | 1271 | 1264 | 32 | 9 | 0.006 | 7.582 |
| 30 | 075844-076421 | *SSR* | 35 | 582 | 182 | 10 | 8 | 0.00936 | 1.704 |
| 31 | 078043-079201 | *rpl16* | 12 | 1163 | 1098 | 30 | 7 | 0.00638 | 7 |
| 32 | 079522-080799 | *rps19-rpl22* | 15 | 1294 | 1178 | 54 | 12 | 0.01014 | 11.943 |
| 33 | 089573-089926 | *SSR* | 22 | 355 | 352 | 2 | 3 | 0.00114 | 0.403 |
| 34 | 101557-103548 | *ndhF* | 15 | 1988 | 1757 | 80 | 13 | 0.01026 | 18.029 |
| 35 | 111153-112227 | *ndhA* | 25 | 1080 | 893 | 50 | 14 | 0.01113 | 9.943 |
| 36 | 134338-134671 | *trnH-psbA* | 36 | 344 | 110 | 7 | 7 | 0.01928 | 2.121 |
