# Supplementary material for: DNA barcoding of Oryza: conventional, specific, and super barcodes
Source: Plant Mol Biol. 2020 Sep 3;105(3):215–28. doi: 10.1007/s11103-020-01054-3 (PMC7858216; doi:10.1007/s11103-020-01054-3)
Supplement: Supplementary file 10 — Supplementary material 10 (DOCX 17.6 kb) [file 11103_2020_1054_MOESM10_ESM.docx]

**A Brief Taxonomic Treatment of *Oryza***

**Oryza** L., Sp. Pl. 1:333, 1753.

1. **O. australiensis** Domin. in Biblioth. Bot. 85(1):333, 1915. Genome type: **EE**.
2. **O. brachyantha** A. Chev. et Roehr. in Compt. Rend. Hebd. Seances Acad. Sci. 159:561, 1914. Genome type: **FF**.
3. **O. coarctata** Roxb., Fl. Ind. (ed. 1932) 2:206, 1832. Genome type: **HHKK**.

*= Porteresia coarctata* (Roxb.) Tateoka in Bull. Natl. Sci. Mus. 8:406, 1965.

1. **O. eichingeri** Peter in Repert. Spec. Nov. Regni Veg. Beih. 40 (1, Anhang):74-75, t. 14, f. 5, 1929. Genome type: **CC**.
2. **O. glaberrima** Steud., Sys. Pl. Glumac. 1:3, 1855. Genome type: **AA**.

*= O. barthii* A. Chev. in Bull. Mus. Natl. Hist. Nat. 16(7):405, 1911.

1. **O. glumipatula** Steud., Syn. Pl. Glumac. 1:3, 1853. Genome type: **AA**.

*= O. longistaminata* A. Chev. et Roehr. in Compt. Rend. Acad. Sci., ser. 2, Mec. Phys. Chim. Sci. Univers. Sci. Terre 159:561, 1914.

1. **O. grandiglumis** (Doell) Prod., Bot. Arch. 1:233, 1922. Genome type: **CCEE**.

*= O. alta* Swallen in Publ. Carnegie Inst. Wash. 461:156, t. 1-2, 1936.

1. **O. latifolia** Desv. in J. Bot. Agric. 1:77, 1813. Genome type: **CCEE**.
2. **O. longiglumis** Jansen in Reinwardtia 2(2):312, f. 13b, 1953. Genome type: **HHJJ**.
3. **O. meridionalis** N. Q. Ng in Bot. J. Linn. Soc. 82(4):328, 1981. Genome type: **AA**.
4. **O. meyeriana** (Zoll. & Moritzi ex Steud.) Baill., Hist. Pl. 12:166, 1893. ≡ *Padia* *meyeriana* Zoll. & Moritzi ex Steud., Syst. Verz. 103, 1846. Genome type: **GG**.

*= O. granulata* Nees et Arn. ex Watt in Dict. Econ. Prod. India 5:500, 1891.

1. **O. minuta** J. S. Presl. et C. B. Presl. in Reliq. Haenk. 1(4-5):208, 1830. Genome type: **BBCC**.

*= O. malampuzhaensis* Kishn. et Chandras in Sci. & Cult. 23:310, 1957.

1. **O. nivara** Sharma et Shastry in Indian J. Genet. Pl. Breed. 25:161, 1965. Genome type: **AA**.

*= O. sativa* L. subsp. *indica* S. Kato in J. Dept. Agric. Kyushu Imp. Univ. 2:275, 1930.

1. **O. neocaledonica** Morat in Bull. Mus. Natl. Hist. Nat., ser. 4, misc. 16(1):2, f. 1, 2, 3-2, 4-2, 4-5, 5, 1994. Genome type: **GG**.
2. **O. officinalis** Wall ex Watt, Dict. Econ. Prod. India 5:501, 1891. Genome type: **CC**.
3. **O. punctata** Kotschy ex Steud., Syn. Pl. Glumac. 1:3, 1855. Genome type: **BB**.
4. **O. rhizomatis** D. A. Vaughan in Bot. J. Linn. Soc. 103(2):160, f. 1, 1990. Genome type: **CC**.
5. **O. ridleyi** Hook f., Fl. Brit. India 7(21):93, 1897. Genome type: **HHJJ**.
6. **O. sativa** L., Sp. Pl. 1:333, 1753. Genome type: **AA**.

*= O. sativa* L. subsp. *japonica* S. Kato in J. Dept. Agric. Kyushu Imp. Univ. 2:275, 1930.

*= O. rufipogon* Griff., Not. Pl. Asiat. 3:5, pl. 144, f. 2, 1851.

1. **O. schlechteri** Pilger in Bot. Jahrb. Syst. 52(1-2):168, 1914. Genome type: **HHKK**.
2. **O. schweinfurthiana** Prod., Bot. Arch. 1:231, 1922. Genome type: **BBCC**.
